# Hsp10 nuclear localization and changes in lung cells response to cigarette smoke suggest novel roles for this chaperonin

**DOI:** 10.1098/rsob.140125

**Published:** 2014-10-29

**Authors:** Simona Corrao, Rita Anzalone, Melania Lo Iacono, Tiziana Corsello, Antonino Di Stefano, Silvestro Ennio D'Anna, Bruno Balbi, Mauro Carone, Anna Sala, Davide Corona, Anna Maria Timperio, Lello Zolla, Felicia Farina, Everly Conway de Macario, Alberto J. L. Macario, Francesco Cappello, Giampiero La Rocca

**Affiliations:** 1 Istituto Euro-Mediterraneo di Scienza e Tecnologia (IEMEST), Palermo, Italy; 2 Dipartimento di Biomedicina Sperimentale e Neuroscienze Cliniche, Università degli Studi di Palermo, Palermo, Italy; 3 Laboratorio di Citoimmunopatologia dell'apparato cardio-respiratorio, Fondazione 'S. Maugeri' IRCCS, Istituto di Veruno, Veruno (NO), Italy; 4 HSR Giglio, Cefalù (PA), Italy; 5 Divisione di Pneumologia, Fondazione 'S. Maugeri' IRCCS, Istituto di Veruno, Veruno (NO), Italy; 6 Fondazione ‘S. Maugeri’ IRCCS, Istituto Scientifico di Cassano delle Murge, Cassano delle Murge (BA), Italy; 7 Dipartimento STEBICEF, Istituto Telethon Dulbecco c/o Universita’ degli Studi di Palermo, Sezione di Biologia Cellulare, Palermo, Italy; 8 Department of Biology and Ecology “La Tuscia” University, Viterbo, Italy; 9 Department of Microbiology and Immunology, School of Medicine, University of Maryland at Baltimore, Baltimore, MD, USA; 10 IMET, Columbus Center, Baltimore, MD, USA

**Keywords:** Hsp10, COPD, bronchial epithelial cells, lung fibroblasts, nuclear localization

## Abstract

Heat-shock protein (Hsp)10 is the co-chaperone for Hsp60 inside mitochondria, but it also resides outside the organelle. Variations in its levels and intracellular distribution have been documented in pathological conditions, e.g. cancer and chronic obstructive pulmonary disease (COPD). Here, we show that Hsp10 in COPD undergoes changes at the molecular and subcellular levels in bronchial cells from human specimens and derived cell lines, intact or subjected to stress induced by cigarette smoke extract (CSE). Noteworthy findings are: (i) Hsp10 occurred in nuclei of epithelial and lamina propria cells of bronchial mucosa from non-smokers and smokers; (ii) human bronchial epithelial (16HBE) and lung fibroblast (HFL-1) cells, *in vitro*, showed Hsp10 in the nucleus, before and after CSE exposure; (iii) CSE stimulation did not increase the levels of Hsp10 but did elicit qualitative changes as indicated by molecular weight and isoelectric point shifts; and (iv) Hsp10 nuclear levels increased after CSE stimulation in HFL-1, indicating cytosol to nucleus migration, and although Hsp10 did not bind DNA, it bound a DNA-associated protein.

## 2. Introduction

The 10 kDa heat-shock protein (Hsp10) is classically considered a mitochondrial co-chaperonin that interacts with Hsp60 to assist in the folding of other mitochondrial proteins [1]. Hsp10 does not contain a mitochondrial targeting sequence, but its translocation to the organelle is mediated by its N-terminal sequence that can form an amphipathic alpha helix, which enables it to cross the mitochondrial membrane [2,3].

Hsp10 has been found in extra-mitochondrial sites such as secretory granules from various cell types [4,5] and in circulation. In the latter location, Hsp10 has been called early pregnancy factor (EPF) because it appears in the maternal serum within 24 h after fertilization in several mammalian species [6–10].

The nuclear gene encoding Hsp10 is *HSPE1* (GeneID: 3336) and it is localized on 2q33.1, head-to-head on opposite strands with the gene of Hsp60 (*HSPD1*) [11]. These genes are controlled by a bidirectional promoter and the regulation of the two genes was found to be simultaneous in eukaryotic cells both under normal conditions and after stress [11].

A number of studies showed that the levels of Hsp10 are increased, usually together with those of Hsp60, in various pathologic conditions such as colon cancer [12,13], post-ischaemic brain [14] and myocardial ischaemia [15,16]. Also, Hsp10 levels were found decreased in other pathological conditions, for example, bronchial carcinogenesis [17]. We also found that Hsp10 and Hsp60 decrease in bronchial dysplasia and adeno-squamous carcinoma [17,18], but increase in airways mucosa in smokers with chronic obstructive pulmonary disease (COPD) [19].

COPD is a chronic inflammatory disease of central and peripheral airways and lung parenchyma characterized by an increased number of tissue lymphocytes, macrophages and neutrophils [20]. Hsps have protective roles intracellularly but are potentially pathogenetic when released outside cells, as they can initiate/perpetuate inflammation [21]. In the lung, intracellular Hsps have predominantly a cytoprotective effect [22–24]. By contrast, extracellular Hsps may act as signal molecules for the immune system, modulating the secretion of proinflammatory cytokines [25–28]. In previous work, we investigated in airways tissues the presence and levels of various Hsps, including Hsp10 and Hsp60, in relation to the COPD severity [19]. We found that Hsp10 and Hsp60 levels were increased in the bronchial epithelium of severe/very severe COPD compared with control non-smokers; by contrast, in lamina propria the number of Hsp10 positive cells was significantly increased in all stages of stable COPD compared with control smokers with normal lung function and non-smoking subjects, while the number of Hsp60-positive cells was significantly higher only in severe/very severe COPD compared with control smokers with normal lung function. Hence, we speculated that both molecules can be involved in maintaining the inflammatory status. However, the different correlations of Hsp60 and Hsp10 levels with smoking habits led us to wonder about the effects of cigarette smoke on the expression and subcellular localization of these proteins in bronchial mucosa, i.e. in epithelial and lamina propria cells. We addressed these issues pertaining to Hsp10 in the work reported here, by means of *in vivo* and *in vitro* experiments.

## 3. Material and methods

### 3.1. Recruitment of subjects, lung function tests, fibreoptic bronchoscopy, collection and processing of bronchial biopsies in patients with COPD

We selected and studied bronchial biopsies from 19 subjects with normal lung function, nine current smokers (age = 64 ± 8; M : F = 8 : 1) and 10 non-smokers (age = 65 ± 9; M : F = 9 : 1). All subjects were Caucasians and were recruited from the Respiratory Medicine Unit of the ‘Fondazione Salvatore Maugeri’ (Veruno, Italy). The severity of the airflow obstruction was staged using current GOLD (Global Initiative for Chronic Obstructive Lung Disease) criteria, as described [19].

Pulmonary function tests were performed as previously described [20]. Pulmonary function tests included measurements of FEV1 and FEV1/FVC under baseline conditions in all the subjects examined (6200 Autobox Pulmonary Function Laboratory; Sensormedics Corp., Yerba Linda, CA, USA).

Subjects were at the bronchoscopy suite at 08.30 h after having fasted from midnight and were pre-treated with atropine (0.6 mg IV) and midazolam (5–10 mg IV). Oxygen (3 l min^−1^) was administered via nasal prongs throughout the procedure and oxygen saturation was monitored with a digital oximeter. A fibreoptic bronchoscope (Olympus BF10 Key-Med, Southend, UK) was passed through the nasal passages into the trachea under local anaesthesia with lidocaine (4%) to the upper airways and larynx. More lidocaine (2%) was sprayed into the lower airways, and four bronchial biopsy specimens were taken from segmental and sub-segmental airways of the right lower and upper lobes using size 19 cupped forceps. Bronchial biopsies for immunohistochemistry were gently extracted from the forceps and processed for light microscopy as previously described [20]. Two samples were embedded in Tissue Tek II OCT (Miles Scientific, Naperville, IL, USA), frozen within 15 min in isopentane pre-cooled in liquid nitrogen and stored at –80°C. The best frozen sample was then oriented and 6 mm thick cryostat sections were cut for immunohistochemical light microscopy analysis and processed as described below.

### 3.2. Immunohistochemistry and scoring system

Two sections from each sample were stained applying immunohistochemical methods with antibodies specific for Hsp10 (rabbit anti-Cpn10 polyclonal antibody, StressGen Biotechnologies, Victoria BC, Canada, Cat. No. SPA-110, dilution 1 : 300) and Hsp60 (mouse anti-Hsp60 monoclonal antibody, Sigma, St. Louis, MO, Cat. No. H4149, dilution 1 : 300). Briefly, after blocking non-specific binding sites with serum derived from the same animal species as that of the secondary antibody, primary antibody was applied at the set dilutions in Tris-buffered saline (0.15 M saline containing 0.05 M Tris–HCl at pH 7.6) and incubated 1 h at 23°C in a humid chamber. Antibody binding was demonstrated with secondary antibodies, anti-rabbit (Vector, BA 1000) or anti-mouse (Vector, BA 2000), followed by ABC kit AP AK5000, Vectastain, and fast-red substrate (red colour). Slides were included in each staining run with human tonsil or nasal polyp as a positive control. For the negative control slides, normal goat, mouse or rabbit non-specific immunoglobulins (Santa Cruz Biotechnology, Santa Cruz, CA, USA) were used at the same protein concentration as the primary antibody.

Morphometric measurements were performed with a light microscope (Leitz Biomed, Leica Cambridge, UK) connected to a video recorder linked to a computerized image system (Quantimet 500 Image Processing and Analysis System, Software Qwin V0200B, Leica). Immunopositivity was scored using a semi-quantitative approach. Three independent observers (F.C., A.D.S. and B.B.) evaluated the immunohistochemical results and quantified the percentage of positive cells for each specimen both in epithelium and lamina propria, down to 100 mm beneath the epithelial basement membrane. Ten high-power fields were examined in each slide and cell counting was performed at 400× magnification.

### 3.3. Preparation of cigarette smoke extract

Cigarette smoke extract (CSE) was obtained following protocols standardized in our laboratory, using a Buchner flask connected to a system acting as a vacuum-driven apparatus [29]. Two cigarettes without filters were combusted and the smoke was bubbled through 50 ml of serum-free Dulbecco's modified Eagle's medium (DMEM). The obtained suspension was then adjusted to pH 7.4 and filtered through a 0.22 μm pore filter. This medium was defined 100% CSE and was applied to cell cultures at different percentages (1, 2, 5 and 10%) within 20 min from preparation. Exposure of cells to CSE was carried out with an incubation time of 24 h.

### 3.4. Cell cultures and *in vitro* model set-up

All experiments were conducted in triplicate. SV40-transformed human bronchial epithelial (16HBE) and immortalized human fetal lung fibroblast (HFL-1) cell lines were used to set up an *in vitro* model in order to assess their behaviour after CSE exposure. Both cell lines were cultured in 8-well chamber slides and 25/75 cm^2^ tissue culture flasks (BD Falcon). Culture medium was composed of DMEM containing 10% fetal bovine serum (FBS), 100 U ml^−1^ penicillin, 100 µg ml^−1^ streptomycin, 0.25 µg ml^−1^ amphotericin B and 2 mM l-glutamine (all from Invitrogen, Milan, Italy). Cultures were maintained at 37°C in a humidified atmosphere of 5% CO_2_. Cells from passages 17–22 were used for the experiments. After reaching 70% of confluence, cells were starved with serum-free DMEM, supplemented with 100 U ml^−1^ penicillin, 100 µg ml^−1^ streptomycin, 0.25 μg ml^−1^ amphotericin B and 2 mM l-glutamine, for 24 h at 37°C in a humidified atmosphere 5% CO_2_. They were then treated with various doses of CSE for different times, as outlined below.

After culturing, cells grown in chamber slides were washed with phosphate-buffered saline (PBS) and fixed in methanol for 20 min at −20°C. Air-dried slides were then stored at −20°C until use (see ‘Immunocytochemistry and scoring system’).

### 3.5. Cell viability assay

Cell viability after CSE exposure was evaluated by MTT [3-(4,5-dimethylthiazol-2-yl)-2,5-diphenyltetrazolium bromide] assay (Sigma Aldrich). After CSE exposure for 24 h in 24-well plates (Corning, Oneonta, NY, USA), culture medium was replaced by MTT, previously diluted in phenol-red free DMEM. After an incubation period of 3 h, the cell precipitate was solubilized by using acidic absolute isopropanol (HCl 0.04 M). The amount of formazan was evaluated spectrophotometrically by reading A_570_, with background subtraction at 650 nm. Triton X-100 (1% v/v) was used as a positive control for cytotoxicity. Triplicate assays were performed using cells at different culture passages. Non-treated (NT) conditions were used as controls in both cell lines.

### 3.6. Immunocytochemistry and scoring system

Immunocytochemistry (ICC) analyses were performed on 16HBE and HFL-1 cells cultured and treated in 8-well chamber slides (BD Falcon), following previously published protocols [30,31]. After culturing, cells were washed with PBS and fixed in methanol for 20 min at −20°C. Air-dried slides were then stored at −20°C until use. For the ICC procedure, the cells were permeabilized with 0.1% Triton X-100 in PBS and then washed with PBS. To block endogenous peroxidases, cells were treated with 0.3% H_2_O_2_ in PBS and then with 1% FBS for blocking non-specific antigens. Antibodies anti-Hsp10 (rabbit polyclonal, StressGen) and anti-Hsp60 (mouse monoclonal, clone LK1, Sigma Aldrich) were used at 1 : 300 dilution. Antibody anti-prolyl 4-hydroxylase (clone 5B5, Dako, Copenhagen, Denmark) was used at 1 : 100 dilution as positive control for the HFL-1 cells. Antibody anti-cytokeratin 8 (mouse monoclonal, Sigma) was used at 1 : 200 dilution as positive control for the 16HBE cells. Negative controls were obtained subtracting the primary antibody incubation step. The detection system used was the LSAB (Labelled Streptavidin-Biotin)-2 kit (Dako), an avidin–biotin complex system in which a biotinylated secondary antibody reacts with peroxidase-conjugated streptavidin molecules. The 3-amino-9-ethylcarbazole (AEC)+ High Sensitivity Substrate Chromogen Ready-to-Use (Dako Cytomation) was used as developer. Each passage was preceded by washes with PBS. The positive reaction was observed using a light microscope (Leica DM 5000B).

Immunopositivity was scored using a semi-quantitative approach [32,33]. Three independent observers (F.C., R.A. and G.L.R.) separately evaluated the immunocytochemical results in epithelium and lamina propria and determined the percentage of positive cells for each specimen. Ten high-power fields were examined in each culture slide and counting of the cells was performed at 400× magnification.

### 3.7. Resin-embedding procedures and immunogold analysis for Hsp10 subcellular localization

LR-White was used as resin to embed 16HBE and HFL-1 cells. Cell fixation was carried out using a fixative solution (4% paraformaldehyde, 1× PBS pH 7.4, 0.2% glutaraldehyde). After washing with 1× PBS pH 7.4, the cells were dehydrated by successive passages through increasing percentages of ethanol solutions (30, 50, 70 and 80%). Then cells were embedded in LR-White resin. Ultrathin sections of 70–100 nm were layered on gold mesh grids. To block endogenous peroxidases, the grids with sections were incubated with 0.3% H_2_O_2_. To block non-specific antigens, 3% bovine serum albumin (BSA; Aurion BSA-c, Electron Microscope Sciences, Hatfield, PA, USA) in 1× PBS pH 7.6 was used. For immunolocalization, ultrathin section-containing mesh grids were incubated with anti-Hsp10 (rabbit polyclonal, StressGen) and anti-Hsp60 (mouse monoclonal, clone LK1, Sigma Aldrich) at 1 : 30 dilution, and then with 10 nm colloidal gold particle-coupled anti-rabbit (10 nm gold-labelled goat anti-rabbit IgG (H + L) RPN421V, Amersham Biosciences, Milan, Italy) or anti-mouse (Electron Microscope Sciences) secondary antibodies at 1 : 30 dilution.

A fixation step with 2% glutaraldehyde preceded the contrast procedure. The latter was carried out incubating grids with 1% (w/v) uranyl acetate in dark, methanol, and then with Reynold's lead citrate solution in dark. After washing with deionized water and drying, sections were examined with a transmission electron microscope (JEM-1220, JEOL, Milan, Italy).

### 3.8. One-dimensional protein electrophoresis (SDS-PAGE) and western blotting

Measurements were carried out of the variations of Hsp10 and Hsp60 in 16HBE and HFL-1 after treatment with various percentages of CSE (1, 2, 5 and 10%) and compared with control conditions, using one-dimensional (1D) sodium dodecyl sulfate-polyacrylamide gel electrophoresis (SDS-PAGE). A fixed amount of total and sub-fractionated proteins (30 µg for each sample) was loaded with sample buffer (0.5 M Tris–HCl pH 6.8, 10% SDS, 10% glycerol, bromophenol blue, 0.2 M dithioerythritol (DTE)) and applied to a 12% SDS-PAGE gel. Gel run was performed for 2 h at 23°C. For the subsequent western blotting, gels obtained from 1D electrophoresis were incubated in transfer buffer (190 mM glycine, 25 mM Tris, 20% methanol) for 30 min and then blotted on polyvinylidene di-fluoride (PVDF) membrane (Hybond-P, GE Healthcare, Milan, Italy). After antigen blocking for 1 h with 3% BSA in 0.05% Tween-20 Tris-buffered saline (T-TBS) pH 7.6, membranes were exposed overnight at 4°C to specific primary antibodies: anti-Hsp10 (rabbit polyclonal, StressGen) at 1 : 2000 dilution, or anti-Hsp60 (mouse monoclonal, clone LK1, Sigma Aldrich) at 1 : 1000 dilution; anti-β-actin (mouse monoclonal, clone AC-74, Sigma Aldrich) at 1 : 2000 dilution and anti-LDH (rabbit monoclonal, clone EP1563Y Epitomics) at 1 : 2500 dilution were used as cytoplasmic control antibodies and anti-lamin A (rabbit polyclonal, Sigma Aldrich) at 1 : 5000 dilution was used as nuclear control antibody. All antibodies were diluted in antibody buffer (1% BSA, 0.05% T-TBS). Membranes were then incubated with specific horseradish peroxidase (HRP)-labelled secondary antibodies, anti-rabbit (1 : 50 000 dilution) and anti-mouse (1 : 10 000 dilution; both from GE Healthcare), for 1 h. They were exposed to chemo-luminescent substrate (Immobilon, Millipore, Billerica, MA, USA) for 5 min and then developed in a dark room on chemiluminescence films (Hyperfilm, GE Healthcare).

### 3.9. Two-dimensional protein electrophoresis (SDS-PAGE) and western blotting

Total proteins from NT and 5% CSE-treated 16HBE and HFL-1 cells were separated according to two independent properties in two steps, using two-dimensional (2D) electrophoresis. In the first-dimension run, isoelectric focusing (IEF), proteins were separated according to their isoelectric point (pI) on immobilized pH gradient (IPG) strips (Immobiline DryStrip gels, GE Healthcare). In the second-dimension run, SDS-PAGE separated the proteins according to their molecular weight. For this study, we used two 7-cm IPG strips with nonlinear pH gradient: 4–7 for Hsp60 and 6–11 for Hsp10 (GE Healthcare) and a 10% 2D gel for SDS-PAGE. Prior to IEF, each IPG strip for each condition was previously rehydrated with rehydration solution (8 M urea, 2% w/v CHAPS, 0.5% v/v IPG buffer, 0.002% bromophenol blue (1% stock solution), double-distilled water, to which was added the reducing agent dithioerythritol prior to use). Samples were diluted in this solution and allowed to enter IPG strips for 1 h at 23°C. The first-dimension run included six steps at various voltages (‘step and hold’ or ‘gradient’) at different times: (i) hold at 30 V for 720 min, (ii) hold at 100 V for 15 min, (iii) gradient 100 V to 300 V for 30 min, (iv) hold at 300 V for 20 min, (v) gradient 300 V to 3000 V 60 min and (vi) hold at 3000 V for 105 min.

After IEF, protein-separated IPG strips were equilibrated with SDS equilibration solution (6 M urea, 75 mM Tris–HCl pH 6.8, 29.3% glycerol, 2% SDS, double-distilled water), prepared in order to obtain two buffers, one containing DTE as reducing agent (Buffer A) and one containing iodoacetamide (which alkylates reduced sulfurs) and bromophenol blue (Buffer B). Equilibrated IPG strips were used for the second-dimension run, which comprised two subsequent steps at different constant currents and different times: 10 mA gel^−1^ for 10 min; and 20 mA gel^−1^ for 120 min.

For both first- and second-dimension runs the Zoom Dual Power adapter (Life Technologies, Milan, Italy) was used.

### 3.10. Image acquisition and data analysis

Two-dimensional gels were stained with ammoniacal silver nitrate as previously described [34]. Silver-stained gels were imaged with a ChemiDoc MP system (Bio-Rad, Milan, Italy). Gel analysis was then performed using Image Master Platinum software (GE Healthcare). As internal standard for gel calibration, we used an array of features of the cell lysate, whose pI and relative molecular mass (Mr) were formerly estimated by interpolation with serum proteins that had co-migrated with the whole cell lysate. Further identifications of spots of interest were made by western blotting of 2D gels.

### 3.11. Coomassie blue staining, nano-high performance liquid chromatography and electrospray mass spectrometry

Proteins were stained with Coomassie Brilliant Blue G-250 stain (SimplyBlue Safestain, Invitrogen). Stained gels were digitalized, and image analysis was performed using Image Master Platinum software. Spots from 2DE maps of biological interest were carefully excised from the gel and subjected to in-gel trypsin digestion according to published procedures [35] with minor modifications. Protein identifification was performed as previously reported [36] through a nanoHPLC (Proxeon, Bruker Daltonics, Macerata, Italy) and MS/MS ion trap (Amazon ETD, Bruker Daltonics) system. Instrument settings were consistent with our previous studies [37].

The spray capillary was a fused silica capillary, 0.090 mm o.d., 0.020 mm i.d. For all experiments, a sample volume of 15 µl was loaded by the autosampler onto a homemade 2 cm fused silica precolumn (100 µm i.d.; 375 µm o.d.; Reprosil C18-AQ, 5 µm, Dr. Maisch, Ammerbuch-Entringen, Germany). Sequential elution of peptides was accomplished using a flow rate of 300 nl min^−1^ and a linear gradient from Solution A (2% acetonitrile; 0.1% formic acid) to 50% of solution B (98% acetonitrile; 0.1% formic acid) in 40 min over the precolumn in-line with a homemade 15 cm resolving column (75 µm i.d.; 375 µm o.d.; Reprosil C18-AQ, 3 µm, Dr. Maisch). The acquisition parameters for the instrument were as follows: dry gas temperature, 220°C; dry gas, 4.0 l min^−1^; nebulizer gas, 10 psi; electrospray voltage, 4000 V; high-voltage end-plate offset, −200 V; capillary exit, 140 V; trap drive, 63.2; funnel 1 in, 100 V, out 35 V and funnel 2 in, 12 V, out 10 V; ICC target, 200 000; maximum accumulation time, 50 ms. The sample was measured with the ‘Enhanced Resolution Mode’ at 8100 *m*/*z* per second (which allows mono-isotopic resolution up to four charge stages) polarity positive, scan range from *m*/*z* 300 to 1500, five spectra averaged and rolling average of 1. The ‘Smart Decomposition’ was set to ‘auto’.

Acquired CID (collision-induced dissociation) spectra were processed in DataAnalysis v. 4.0, and deconvoluted spectra were further analysed with BioTools v. 3.2 (Bruker Daltonics) software and submitted to Mascot search program (in-house v. 2.2, Matrix Science, London, UK). The following parameters were adopted for database searches with Mascot search program: NCBInr database (release date 10 March 2013; 247 209 sequences); taxonomy = *Homo sapiens*; peptide mass tolerance of ±0.3 Da; fragment mass tolerance of ±0.3 for CID ions; enzyme specificity, trypsin with two missed cleavages considered; fixed modifications: carbamidomethyl (C); variable modifications: oxidation (M).

### 3.12. Total and sub-fractionated proteins extraction

The 16HBE and HFL-1 cells were collected after treatment and were lysed with lysis buffer (20 mM Tris–HCl pH 7.5, 1% Nonidet P-40, 50 mM NaCl, 1× Proteases Inhibitor) in order to obtain total proteins. In parallel, separate nuclear and cytoplasmic fractions were obtained using Nuclear Extract Kit (Active Motif, Carlsbad, CA, USA), following the manufacturer's instructions. Protein concentration was determined spectrophotometrically, using the DC Protein Assay kit (Bio-Rad), following the manufacturer's instructions.

### 3.13. Chromatin extracts and western blotting

Chromatin protein extracts derived from 16HBE and HFL-1 cells were prepared according to previously published protocols [38]. 16HBE and HFL-1 cells were collected and each cell line was split in two tubes with or without 2% formaldehyde in 100 µl of 0.5 × M buffer (10 mM HEPES–KOH (pH 7.6), 25 mM KCl, 5 mM MgCl_2_, 5% glycerol), 1 mM dithiothreitol (DTT) and 1 mM phenyl methyl sulfonyl fluoride (PMSF) and incubated for 15 min at 23°C. Both cross-linked and not cross-linked samples were added with 125 mM glycine and transferred to a microfuge tube containing 100 µl of M buffer, 0.8% NP-40, 1 mM DTT, and 1 mM PMSF; incubated on ice for 15 min; homogenized with a pestle; and pelleted by centrifugation at 2100*g* for 5 min at 4°C. The supernatant (cytoplasmic extracts) was recovered and the pellet (chromatin proteins) was resuspended in boiling SDS-PAGE loading buffer. Proteins were fractionated by SDS-PAGE and analysed by protein blotting using anti-Hsp10 (rabbit polyclonal, StressGen) at 1 : 2000 dilution. The chemiluminescent HRP-conjugated secondary antibodies (anti-rabbit 1 : 50 000 dilution) were used and the reaction developed using the Super Signal West Femto substrate (Pierce, Milan, Italy) and acquired with the ChemiDoc XRS imager (Bio-Rad).

### 3.14. Nucleosome band-shift assay

A total of 146-bp and 251-bp amplified rDNA fragments [39] were incubated with increasing amounts of recombinant human and recombinant *Drosophila* ISWI [40], respectively, in 10 µl final volume containing 50 mM Tris–HCl (pH 8), 50 mM NaCl, 1 mM MgCl_2_, 100 µg ml^−1^ chicken albumin, 0.05% NP-40 and 10% glycerol. The reaction was incubated for 15 min at 25°C and samples were resolved on 1.4% agarose gel in 0.3× Tris–borate–EDTA buffer at 4°C for 50 min as previously reported [39] and detected by EtBr staining.

### 3.15. Statistical analyses

Data were plotted using MS Excel software. Statistical analyses were performed using GraphPadPrism v. 4 software (GraphPad Software, San Diego, CA, USA). The statistical methods used were non-parametric analyses. The significance of the differences in chaperonins levels between control and CSE treatments was assessed by the Kruskal–Wallis test, and significance of differences between different groups was analysed by the Mann–Whitney test. Values were considered significant when *p* ≤ 0.05.

### 3.16. Computational studies (*in silico* analyses)

In order to confirm the variations in Hsp10 subcellular localization on the basis of physical–chemical parameters, *in silico* analyses were carried out using online ExPASy Proteomics Server (http://www.expasy.org). In this site, methods are found that use various algorithms to determine the characteristics of primary amino acid sequences such as physical–chemical features, based for example on electric charges, hydrophobic moment, number of hydrophobic amino acids, etc. We used PSORT for prediction of subcellular localization and of nucleic acid-binding motifs.

## 4. Results

### 4.1. *In vivo* analysis of Hsp10 and Hsp60 levels and cellular localization

**Figure 1.**
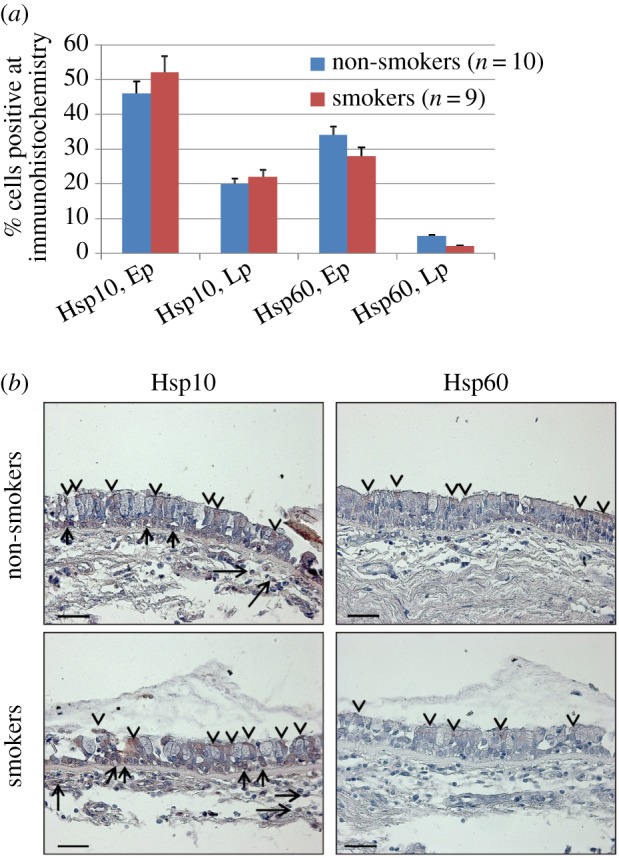
Immunohistochemical detection of Hsp10 and Hsp60 in human bronchial mucosa. (*a*) The levels of Hsp10 in epithelium (Ep) and in lamina propria (L_p_) were similar in non-smokers compared with smokers. Hsp60 was detected only in epithelial cells and its levels were also similar in non-smokers and smokers. Statistical analyses showed that all of the variations were not significant with *p*-values ranging between *p* = 0.1 and 0.4. (*b*) Representative immunohistochemical images. Hsp10 is present in epithelium and lamina propria cells of non-smokers and smokers, whereas Hsp60 is present only in epithelial cells. Arrowheads, epithelial positivity; arrows, lamina propria positivity. Scale bars, 50 µm.

We quantified the levels of Hsp10 and Hsp60 in epithelium and lamina propria of bronchial mucosa of non-smoker and smoker subjects with normal lung function to determine if there was an increase associated with smoking, which is considered a source of stressors for the airways tissues. The clinical characteristics of the subjects studied are shown in the electronic supplementary material, table S1. Hsp10 and Hsp60 levels did not show quantitative changes in epithelium or in lamina propria of the bronchial mucosa of smokers compared with non-smokers (figure 1*a*). Both molecules showed a cytoplasmic positivity, often with a granular pattern resembling a mitochondrial positivity (figure 1*b*). Hsp10 was positive in both groups of subjects, smokers and non-smokers, and both in epithelial and lamina propria cells. Notably, while Hsp60 positivity is clearly outside cell nuclei, the Hsp10 pattern suggests a nuclear positivity which may not be fully explained by a simple superimposition with cytosol.

### 4.2. 16HBE and HFL-1 cell viability after CSE exposure

**Figure 2.**
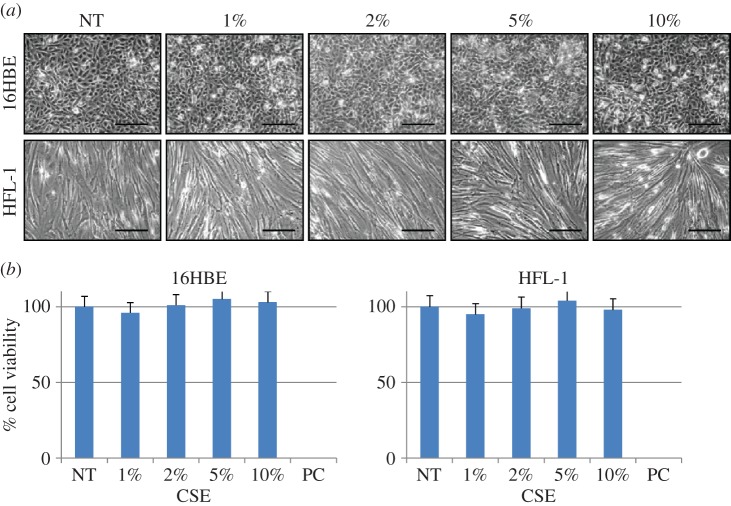
Morphologic and cell viability analyses after cell exposure to CSE. (*a*) Phase-contrast microscopy images of 16HBE and HFL-1 cell lines: non-treated controls (NT) and after 24 h of treatment with CSE at various concentrations from 1 to 10%. No morphologic changes after CSE treatment are apparent, indicating that CSE, at the concentrations used in this work, had no cytotoxic effect. (*b*) Cell viability measured by the MTT test also did not show any cytotoxic effect of CSE, with non-significant differences between the control and treatments (*p* = 0.7). PC, positive control with Triton X-100 incubation. Scale bars, 100 µm.

We performed a set of *in vitro* experiments to determine possible effects on Hsp10 and Hsp60 levels and subcellular localization caused by exposure to CSE. We first determined if CSE exposure at various concentrations caused cell death. HFL-1 and 16HBE cells were incubated for 24 h with CSE at different percentages (1, 2, 5 and 10%) and, after the incubation, the cell morphology was examined by phase-contrast microscopy. For both cell lines, morphological features were maintained after 24 h of treatment (figure 2*a*). Also, to assess the effects of CSE exposure on cell viability, we performed viability assays by using MTT. The doses of CSE used in this study did not produce any detectable decrease in cell viability in HFL-1 or 16HBE cells (figure 2*b*).

### 4.3. Immunolocalization of Hsp10 and Hsp60 in CSE-treated 16HBE and HFL-1 cells

**Figure 3.**
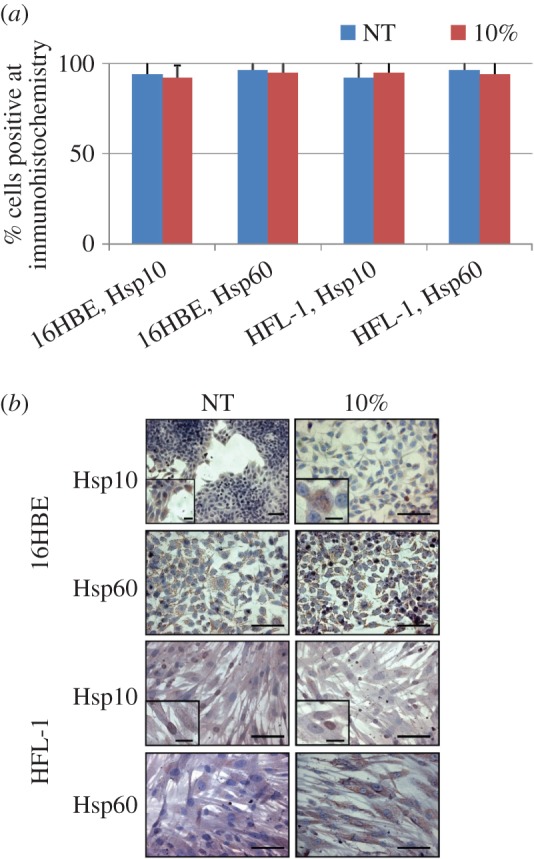
Immunocytochemistry for Hsp10 and Hsp60. (*a*) Levels of Hsp10 and Hsp60 in 16HBE and HFL-1 cells before (NT) and after (10%) incubation with CSE at 10%. No statistically significant differences were found between the groups (*p*-values ranging between 0.48 and 0.68). (*b*) Representative microphotographs showing immunolocalization by immunohistochemistry of Hsp10 and Hsp60 in 16HBE and HFL-1 cells. The insets indicate nuclear localization of Hsp10. Scale bars, 10 µm (figures) and 1 µm (insets).

We quantified the levels of Hsp10 and Hsp60 in 16HBE and HFL-1 cells before and after CSE treatment. Hsp10 and Hsp60 levels did not show quantitative changes after treatment (*p* > 0.05; figure 3*a*). Hsp60 was present in the cytoplasm, often with the granular aspect typical of mitochondrial localization (figure 3*b*). Hsp10 showed not only a wide cytoplasmic, granular distribution but also an appreciable positivity at the nuclear level (figure 3*b*, insets). These data were in agreement with the *in vivo* immunohistochemical results obtained with bronchial mucosa cells as described above, and showed nuclear localization of Hsp10 in treated and untreated cells. To the best of our knowledge, positivity for Hsp10 in the nucleus of airways mucosa cells has never been described before.

### 4.4. Immunogold analysis of Hsp10 and Hsp60 in untreated 16HBE and HFL-1 cells

**Figure 4.**
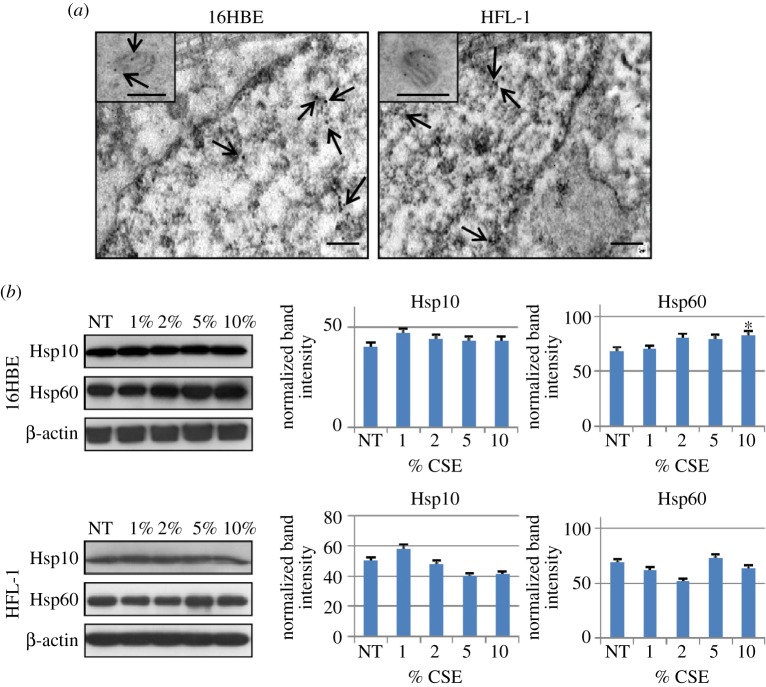
Localization of Hsp10 by electron microscopy and the immunogold technique and western blotting analyses of Hsp10 and Hsp60 in cell lines. (*a*) Immunogold for Hsp10 on 16HBE and HFL-1 cells, showing its nuclear localization in addition to its typical presence in mitochondria (insets). Arrows indicate some of the Hsp10 positivity in the nucleus and in mitochondria. (*b*) Western blotting analyses of total protein extracts from 16HBE and HFL-1 cell lines after treatment with CSE at various concentrations from 1 to 10%, for 24 h, in comparison with NT cells. Statistical analysis was performed using the Mann–Whitney test. Data are represented as mean (±s.e.m.) of three replicate experiments. Only Hsp60 showed a significant increase after 24 h of treatment of 16HBE cells at 10% CSE concentration by comparison with the NT controls (*p < 0.0001). All other variations were not significant, with *p*-values ranging between 0.1 and 0.7.

To verify the immunohistochemical results showing nuclear localization of Hsp10 in normal cells with another method, immunogold analyses were performed on ultrathin sections of untreated 16HBE and HFL-1 cells. Both 16 HBE and HFL-1 cells showed Hsp10 positivity in the nucleus (figure 4*a*), in addition to the typical mitochondrial localization (figure 4*a*, insets). By contrast, we did not find nuclear localization for Hsp60, although both chaperonins were also positive in the cytosol and, slightly, in the plasma membrane in both cell lines.

### 4.5. Western blotting of whole cell lysates

We performed western blotting to further determine if CSE stimulation does or does not induce quantitative changes in Hsp10 and Hsp60 levels; as described above the immunohistochemical method did not detect changes (figure 3*a*). Total proteins extracted from NT 16HBE and HFL-1 cells and after 24 h of CSE exposure were used for 1D SDS-PAGE. Figure 4*b* shows histograms displaying results of semi-quantitative densitometry of Hsp10 and Hsp60 levels in 16HBE and HFL-1 cells after CSE exposure (from 1 to 10%) in comparison with the controls (i.e., cells not treated with CSE). 16HBE expressed both Hsp10 and Hsp60 in all the tested conditions. Hsp60 showed increased levels after 24 h of treatment with 10% CSE by comparison with the untreated controls (*p* < 0.01). By contrast, Hsp10 in 16HBE as well as both molecules in HFL-1 did not show any significant difference between treated and NT cells. The observed difference in the levels of Hsp60 after 24 h of 10% CSE treatment compared with controls is in contrast to the previously described immunocytochemical data, which did not show a significant difference. This discrepancy may be attributed to the higher sensitivity of western blotting as compared with immunohistochemistry for measuring protein levels. In fact, the two methods are complementary with immunohistochemistry having resolution power to map the topography of antigens in subcellular compartments and tissue types, whereas western blotting is advantageous to directly identify immunologically and quantify protein bands. Except for this, the other results from western blotting were in agreement with immunocytochemistry.

### 4.6. Two-dimensional SDS-PAGE on total protein extracts

**Figure 5.**
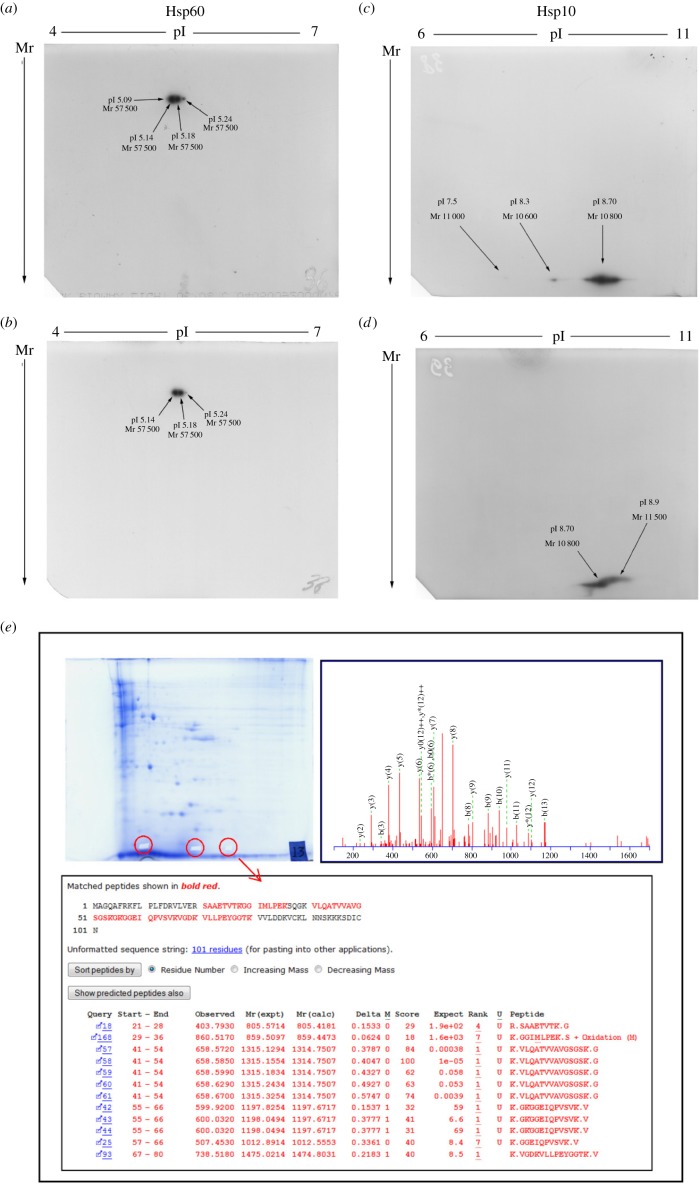
2D SDS-PAGE and mass spectrometry analyses. 2D SDS-PAGE of Hsp60 (*a*,*b*) and Hsp10 (*c*,*d*) before (*a*,*c*) and after (*b*,*d*) treatment of the cells with CSE 5% for 24 h. Both Hsp10 and Hsp60 showed changes in Mr and pI after treatment. (*e*) Proteomic and mass spectrometry analyses confirmed the identity of the three spots in (*c*) as isoforms of Hsp10.

In order to determine if qualitative variations in the Hsp10 and Hsp60 molecules did occur after CSE exposure, 2D SDS-PAGE analyses were performed on 100 µg of total protein extracts. To perform this set of experiments we used two sources of materials for protein extraction, NT cells as control and cells exposed to 5% CSE for 24 h. The choice of this CSE concentration was based on 1D western blot results: we used the highest concentration that did not determine changes in the Hsp10 and Hsp60 levels. Western blots and resulting spots were analysed using gel matching, with reference maps in our laboratory database, as explained in Material and Methods. Figure 5 shows the results of 2D-IPG western blot of the Hsp60 isoforms in the HFL-1 cell line under control condition and after 24 h of treatment with 5% of CSE. Control cells showed four Hsp60 isoforms with the same relative molecular weight (Mr = 57 500) but distinct pIs, 5.09, 5.14, 5.18 and 5.24 (figure 5*a*). After 5% CSE exposure, HFL-1 showed only the absence of the Hsp60 5.09 isoform (figure 5*b*). More differences were instead found when analysing the results of Hsp10 isoforms. Control conditions (figure 5*c*) showed three different isoforms of Hsp10: one, the more highly expressed and basic isoform (likely to be the typical canonical Hsp10), had an apparent Mr of 10 800 and pI of 8.7 (relative to the most expressed isoform); the other two isoforms had a Mr of 10 600, with pI 8.3, and Mr 11 000, with pI 7.5. Considerable modifications were visible following CSE treatment. On the one hand, the canonical isoform showed Mr and pI shifts (Mr = 11 500; pI = 8.9), on the other hand, the two acid forms became undetectable after treatment (figure 5*d*). Very similar results were obtained for 16HBE cells (data not shown). Hence, CSE exposure in bronchial cell lines induced qualitative modifications in the two chaperonins, particularly in Hsp10.

### 4.7. Mass spectrometry analyses

In order to further verify the identity of the three Hsp10 isoforms described in the preceding subsection, we carried out mass spectrometry analyses on spots cut from Coomassie blue-stained 2D gels. After trypsin digestion and HPLC analyses, identification of amino acid sequences of the spots was obtained by ESI-MS (electrospray ionization-mass spectrometry). Recognized peptide fragments possessed amino acid sequences comprising the primary amino acid sequence of Hsp10 (figure 5*e*), confirming the identity of the basic spot as canonical Hsp10. Also, the tryptic digest of the other two more acidic spots excised from the gels showed the presence of peptide fragments belonging to the amino acid sequence of Hsp10, therefore identifying also these two spots as acid isoforms of Hsp10. The peptide fragment recognized in all the three spots was ^41^VLQATVVAVGSGSK^54^. The complete list of peptides identified by MS experiments is reported in the electronic supplementary material, table S2. These findings confirm the 2D western blotting results and support the idea that CSE treatment might be responsible for the decrease or disappearance of Hsp10 acid isoforms. Moreover, CSE treatment may have caused post-translational modifications responsible for the shifts in Mr and pI of the canonical isoform of Hsp10.

### 4.8. Western blotting analysis for Hsp10 in subcellular fractions of 16HBE and HFL-1 cells

**Figure 6.**
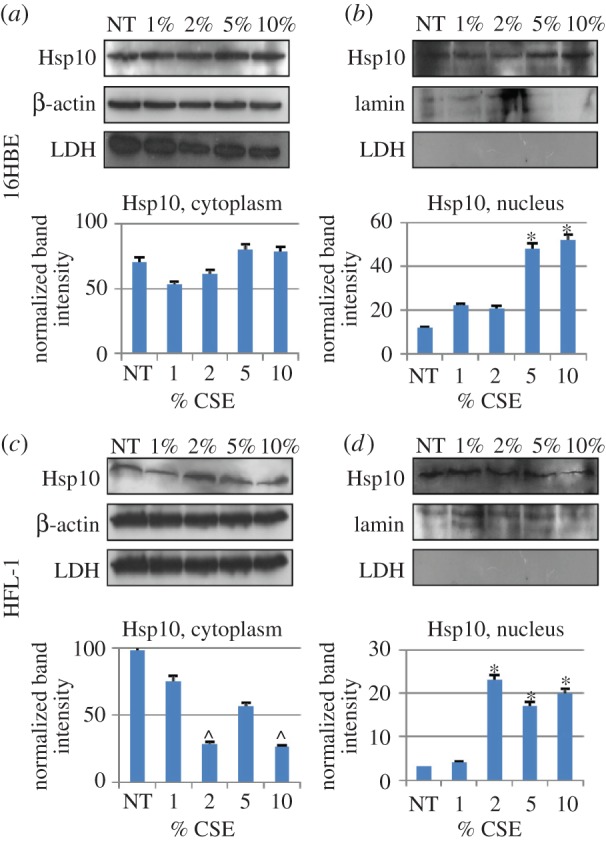
Identification of Hsp10 in subcellular fractions by western blotting. Hsp10 was detected in the cytoplasmic fraction (*a*,*c*) of 16HBE (*a*) and HFL-1 (*c*) cells treated with various concentrations of CSE (from 1 to 10%, as indicated). Likewise, Hsp10 was detected in the nuclear fraction of both cells lines (*b*,*d*) and its levels were significantly higher after treatment with 2% or higher concentrations of CSE by comparison with NT cells in HFL-1 (*p* < 0.05), and 5% or higher in 16HBE (*p* < 0.05), indicating that this treatment induced an increase in Hsp10 in the nucleus. A significant (*p* < 0.05) decrease in Hsp10 was observed in the cytoplasm of HFL-1 cells after treatment with 2 and 10% CSE. β-Actin and lamin were used as cytoplasmic and nuclear internal controls, respectively. Possible contamination of nuclear preparations with cytoplasm was monitored by testing for lactate dehydrogenase (LDH), but no contamination was detected.

In order to verify Hsp10 levels in the cytoplasm and in the nuclear fraction, we performed western blotting analyses on cytoplasmic and nuclear protein extracts, sub-fractionated from control and 24 h-CSE-treated 16HBE and HFL-1 cells. In both cells, Hsp10 was found in all tested conditions, not only in the cytoplasmic but also in the nuclear fraction (figure 6). Cytoplasmic contamination of the nuclear fraction was searched by assessing lactate dehydrogenase, and the results indicated no contamination. Interestingly, the statistical analyses showed an increase in Hsp10 levels in the nucleus after 5%, and 10% CSE stimulation in 16HBE and after 2, 5 and 10% CSE stimulation in HFL-1. By contrast, cytosolic Hsp10 was reduced after 2% and 10% CSE stimulation in HFL-1 but did not change in 16HBE. These data suggest a subcellular redistribution of Hsp10, at least in HFL-1 cells, after CSE exposure, i.e. in these cells Hsp10 seems to migrate from cytosol to nucleus.

### 4.9. Western blotting analyses of Hsp10 on chromatin proteins and gel retardation assays

**Figure 7.**
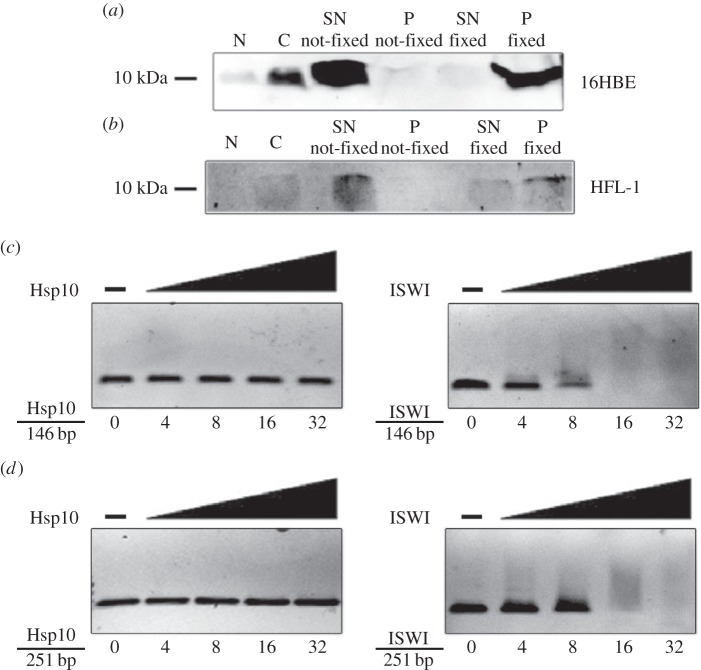
Search for Hsp10 interactors in the nucleus. 1D western blotting analyses of Hsp10 on chromatin proteins and gel retardation assays (band shift) on 16HBE (*a*) and HFL-1 (*b*) cells. SN not-fixed, supernatant fraction not-fixed with formaldehyde; P not-fixed, chromatin fraction not-fixed with formaldehyde; S fixed, supernatant fraction fixed with formaldehyde; P fixed, chromatin fraction fixed with formaldehyde. Gel retardation assays were carried out on 0.5 nmol of 146 bp (*c*) and 251 bp (*d*) rDNA fragments, using increasing amounts (0, 4, 8, 16 and 32 nmol) of recombinant human Hsp10 and recombinant *Drosophila* ISWI used as control.

To better understand the significance of Hsp10 localization in the nucleus in normal conditions, we performed a 1D SDS-PAGE on supernatant (SN) and chromatin pellet (P) from not-fixed and fixed 16HBE and HFL-1 cells in control condition (not exposed to CSE). Fixation was performed using formaldehyde. Figure 7*a*,*b* shows the results of the western blotting for Hsp10. Both cell lines that were not fixed with formaldehyde showed the presence of Hsp10 in the supernatant only (SN not-fixed), but the chaperonin was not present in not-fixed chromatin (P not-fixed). The cells that were fixed (both cell lines), showed the absence of Hsp10 in the supernatant (S fixed) and its presence in the chromatin faction (P fixed). These results show that Hsp10 in the nucleus is close to the chromatin.

In order to determine if Hsp10 would directly interact with DNA, gel retardation assays (band-shift assays) were carried out on 0.5 nmol of 146- and 251-bp rDNA fragments (50 and 80 ng, respectively), using increasing amounts (0, 4, 8, 16, and 32 nmol) of recombinant human Hsp10 and, as control, recombinant *Drosophila* ISWI. No band shift was observed with human recombinant Hsp10, neither on 146-bp (figure 7*c*) nor on 251-bp (figure 7*d*) rDNA fragments, in contrast to results with *Drosophila* recombinant ISWI. These findings indicate that Hsp10 does not directly interact with DNA, but is bound to chromatin, suggesting that the chaperonin interacts with nuclear proteins whose identity remains to be clarified.

### 4.10. *In silico* investigations and computational analyses for protein sorting

In order to find structural features that would explain the results obtained *in vitro*, we performed *in silico* analyses, using available tools to characterize the Hsp10 molecule. ExPASy Server tools were used for detecting physical–chemical features. All the algorithms based their analyses on primary amino acid sequences, charge distribution and other molecular parameters, but did not take into account three-dimensional structures and/or physical–chemical variations with respect to a physiological *in vivo* environment. By using PSORT II, prediction of protein subcellular localization was investigated for Hsp10. PSORT II uses a series of methods taking into account several physical–chemical parameters, for instance number of basic amino acids, hydrophilic state, hydrophobic moment and other features. Among these methods, the *k*-Nearest Neighbors is an algorithm that evaluates the probability of sub-localization in different cell compartments. Table 1 shows the results, predicting a predominant cytoplasmic localization (56.5%) for Hsp10, which is remarkable considering that Hsp10 is classically thought to be a mitochondrial protein. The nucleus was predicted as second subcellular localization (17.4%), which supports our experimental results, indicating presence of Hsp10 in the nucleus.

**Table 1.** Predicted subcellular localization for Hsp10. *k*-Nearest Neighbors (PSORT II) results, showing the prediction of the possible subcellular localization of Hsp10.

cell compartment	Hsp10 (%)
cytoplasmic	56.5
mitochondrial	8.7
nuclear	17.4
cytoskeletal	8.7
peroxisomal	8.7

### 4.11. Prediction of localization signals and nucleic acid-binding motifs

In order to find possible explanations for the presence of Hsp10 in the nucleus, PSORT II software was used to predict the presence of an amino acid sequence that could serve as a DNA- or RNA-binding motif. Data in the electronic supplementary material, table S3 show that Hsp10 does not possess typical DNA- or RNA-binding motifs. This result indicates that Hsp10 in the nucleus does not bind DNA but a chromatin protein, as indicated by the experimental results described above.

## 5. Discussion

Hsp10 and Hsp60 have for a long time been considered typical intramitochondrial molecules devoted to assisting protein folding inside the organelle. However, there is now evidence that the Hsp10 and Hsp60 are also localized in other cell compartments such as cytosol, granules and plasma membrane [3]. The subcellular localization of Hsp10 was investigated in rat tissues by the immunogold technique. In all examined anatomical regions, Hsp10 was present in mitochondria, but a strong and specific Hsp10 positivity was also found in several extra-mitochondrial compartments in various structures. These included zymogenic granules in pancreatic acinar cells, growth hormone granules in anterior pituitary and pancreatic polypeptide granules in pancreatic islet cells [41]. It was proposed that Hsp10 may reach the bloodstream via these secretory granules. It was also reported that Hsp10 labelling in these extra-mitochondrial compartments was at least comparable with, if not higher than, that seen in mitochondria. By contrast, the labelling in other cell compartments such as the nucleus was generally weak, near background levels. These observations for Hsp10 were similar to those that had been reported earlier for Hsp60 [42].

The role of Hsp10 residing in the cytoplasm is not yet well understood. It was demonstrated that an interaction of Hsp10 and Hsp60 with procaspase-3 occurs in the mitochondria of Jurkat cells, and that the disruption of this complex is accompanied by the release of active caspase fragments from the mitochondrial intermembrane space, which is followed by progression to cell death [43]. It was also demonstrated that the increase in Hsp10 levels in cardiac myocytes protects mitochondrial function, thus exerting an anti-apoptotic effect during ischaemia/reoxygenation [16]. Hsp10 and Hsp60 have a similar distribution and higher than normal levels in cells before and after stress and during pathogenesis of a number of diseases, including cancer [44] and inflammatory diseases [45]. Furthermore, Hsp10 has also been found increased in some precursors of normal human bone-marrow cells and it disappeared during lineage maturation, while Hsp60 did not show changes in levels during bone marrow cell differentiation [46]. These data support the notion that intracellular Hsp10 has roles, probably independent of Hsp60, different from its canonical function as co-chaperone for Hsp60 in protein folding inside mitochondria.

To the best of our knowledge, this is the first report of Hsp10 localization in nucleus of human bronchial cells. The work reported here shows that (i) in specimens from subjects with normal respiratory function, either smokers or non-smokers, Hsp10, in contrast to Hsp60, was localized not only in the cytoplasm but also in nuclei of epithelial and lamina propria cells of bronchial mucosa *in vivo*. The Hsp10 levels in the nucleus was the same in the two groups of subjects. (ii) Human bronchial epithelial cells and lung fibroblasts, *in vitro*, showed Hsp10 but not Hsp60 positivity in the nucleus, before and after CSE exposure; (iii) CSE did not cause an appreciable overall increase in levels of Hsp10 in either cell line; (iv) CSE exposure determined in both cells lines qualitative changes in Hsp10, as indicated by Mr and pI shifts. In addition, the less expressed isoforms of Hsp10 were undetectable after CSE exposure; (v) Hsp10 nuclear levels increased after CSE exposure in HFL-1, suggesting a migration of this protein from cytosol to nucleus in response to the exogenous stimulus; (vi) Hsp10 in the nucleus did not bind DNA but it bound one or more nuclear proteins; and (vii) bioinformatics predicted that Hsp10 can occur in the nucleus but it does not have structural features, i.e. motifs, for DNA binding.

In a previous paper, we discussed three molecules, EPF, extracellular Hsp10, and intracellular Hsp10, all of which had been described independently, at different times, with diverse functions [1]. These three molecules are in fact variants of the product of the same gene. How these variants originate and what are their distinctive structural features, if any, is still unclear, but investigations in this area should provide data useful to understand the various functions of Hsp10 and the mechanisms pertaining to its various localizations.

The results from the proteomics experiments provided novel insights on the existence of different isoforms of human Hsp10, which are differently regulated by a stressor such as CSE. Our data demonstrated that in normal cell lines three different isoforms of Hsp10 are detectable (centered at pI of 7.5, 8.3 and 8.7), with the most basic one being the most expressed (or canonical) form. A database search in Expasy and related databases (not shown) showed that the only experimental evidence of human Hsp10 (UniProtKB accession number P61604) in a 2D database refers to a spot at pI = 8.9, identified by researchers of the University College Dublin (http://proteomics-portal.ucd.ie:8082/cgi-bin/2d/2d.cgi?P61604) in human brain and heart samples run on 6–11 IPG strips. However, the reported Mr of this molecule is between 8280 and 8900 Da. Given a theoretical pI of 8.9 and a theoretical Mr of 10 800 Da, it seems unclear which modification in the mature protein may have caused this shift. No data are available, to the best of our knowledge, on the existence of other isoforms of Hsp10 at more acidic pI values, such as those we demonstrated in the two cell lines we investigated by means of both 2D western blotting and tandem mass spectrometry. We can hypothesize that these isoforms are derived by post-translational modifications occurring on the canonical Hsp10 molecule. Database searches showed that one phosphorylation site has been demonstrated at threonine 79 [47], and another four sites are predicted to exist at serine and tyrosine residues (NetPhos 2.0 analysis, not shown). Phosphorylation, albeit leading to nearly undetectable Mr changes at the protein level, may have a dramatic effect on pI: for example, Scansite analysis of Hsp10 showed that a single phosphorylation event may result in an isoform with a theoretical pI of 7.38 (http://scansite.mit.edu). Moreover, multiple acetylation sites have been demonstrated at lysine and alanine residues in the same protein sequence, apart from the known N-terminal acetylation (at alanine). All of these modifications may have relevant effects on the pI of the modified protein, as well as on its molecular weight. Impairment of deacetylation enzymes, which may add further complexity to the isoform pattern of Hsp10, has been linked to cigarette smoke stimulus in lung cells, as demonstrated for histone deacetylases (HDACs) and sirtuins, whose impairment has been correlated to the inflammatory stimulus arising from smoke challenge [48–52].

The demonstration of the exact modifications occurring to the more acidic isoforms of Hsp10 and the identification of the cellular modifying enzymes is beyond the scope of the present paper, but it is currently under active investigation in our laboratory.

Data reported here indicate that in human cells of the respiratory mucosa there are at least three different intracellular locales for Hsp10: mitochondrial, nuclear, and cytosolic. It is likely that in these three locations there occur as many variants of Hsp10 with distinct roles, perhaps all related to chaperoning functions but pertaining to distinct substrates and/or protein quality control pathways. The balance between these forms is probably well regulated in the cell but this is still a matter for further research. One can hypothesize that the induction of Hsp10 gene expression, or overexpression, by a variety of stressors results in a redistribution of the chaperonin whose final destination and functions depends on the gene-inducing cause and mechanism. Thus, Hsp10 can enter into the mitochondria to collaborate with Hsp60 in protein folding, or transfer to secretory granules (either for chaperoning proteins in them or for escaping out of the cells and reaching the bloodstream), or translocate to the nucleus.

Hsp10 could translocate into the nucleus by itself via an as yet to be discovered mechanism, or translocation could take place by association with another protein equipped for that task, thus enabling the chaperonin lacking a nuclear localization signal to gain the intranuclear space. In the latter location, Hsp10 may play roles such those expected from intranuclear molecules, e.g. participation in cell proliferation, differentiation and death.

The electronic supplementary material, table S4 shows the results of an *in silico* analysis to search for known interactors of Hsp10 with clear nuclear localization. Twenty-two unique molecules have been discovered using both Biogrid and IntAct databases. Of note, some of these molecules, are known transcription factors, or molecules involved in the control of the cell cycle, or even other chaperone molecules, such as mortalin. In addition, other interactors of Hsp10 have been shown to have direct and indirect roles in chromatin remodelling and transcriptional control. For example, CBX3 recognizes and binds histone H3 tails methylated at ‘Lys-9’. This may lead to epigenetic repression or, as recently shown, also to differential maturation of RNAs (co-transcriptional alternative splicing) [53]. Also, the histone-binding protein RBBP7 is a component of several complexes that regulate chromatin metabolism. Another relevant molecule is ATF2: it is a transcriptional activator that regulates the transcription of genes such as those involved in anti-apoptosis, cell growth and DNA damage response. In particular, recent data linked ATF2 downregulation to an increased apoptosis after oxidative stress stimuli [54].

Finally, variations in the levels of Hsp10 beyond the normal range for each localization may have a pathogenic significance. For instance, the increase, or decrease, of Hsp10 in cancer could be involved in regulation of apoptosis of tumour or pre-tumoural dysplastic cells.

## Supplementary Material

Supplementary Table 1

## Supplementary Material

Supplementary Table 2

## Supplementary Material

Supplementary Table 3

## Supplementary Material

Supplementary Table 4
