# Supplementary material for: Hsp10 nuclear localization and changes in lung cells response to cigarette smoke suggest novel roles for this chaperonin
Source: Open Biol. 2014 Oct 29;4(10):140125. doi: 10.1098/rsob.140125 (PMC4221893; doi:10.1098/rsob.140125)
Supplement: Supplementary Table 1 [file rsob140125supp1.docx]

Supplementary table 1: Clinical characteristics of the subjects studied by immunohistochemistry

|  | Control  non-smokers | Control smokers  normal lung function |
| --- | --- | --- |
| Number | 10 | 9 |
| Age (years) | 65±9 | 64±8 |
| M/F | 9:1 | 8:1 |
| Pack years | 0 | 41±27 |
| Ex/current smokers | 0 | 1/8 |
| FEV1 pre-β2 (% predicted) | 115±13 | 103±14 |
| FEV1/FVC (%) | 84±9 | 80±7 |
