# Supplementary material for: Hsp10 nuclear localization and changes in lung cells response to cigarette smoke suggest novel roles for this chaperonin
Source: Open Biol. 2014 Oct 29;4(10):140125. doi: 10.1098/rsob.140125 (PMC4221893; doi:10.1098/rsob.140125)
Supplement: Supplementary Table 2 [file rsob140125supp2.docx]

| **Spot No.** | **Peptides identified by MS/MS** | | | | **Mascot**  **Ion**  **Score** | **Protein Sequence Coverage**  **(%)** | **NCBI Accession Number** | **Protein ID** |
| --- | --- | --- | --- | --- | --- | --- | --- | --- |
|  | **m/z** | **Charge**  **State** | **Start-End** *^a^* | **Sequence** |  |  |  |  |
| 1A | 403.79  860.51  658.57  599.92  507.45  738.51 | 2 +  2 +  2 +  2 +  2 +  2 + | 21-28  29-36  41-54  55-66  57-66  67-80 | R.SAAETVTK.G  K.GGIMLPEK.S Oxidation (M)  K.VLQATVVAVGSGSK.G  K.GKGGEIQPVSVK.V  K.GGEIQPVSVK.V  K.VGDKVLLPEYGGTK.V | 29  18  100  41  40  40 | 55 | gi\|119590561 | heat shock 10kDa protein 1 (chaperonin 10), isoform CRA_b [Homo sapiens] |
| 2A | 658.57 | 2+ | 40-53 | K.VLQATVVAVGSGSK.G | 93 | 14 | gi\|4008131 | chaperonin 10 |
| 3A | 658.57 | 2+ | 40-53 | K.VLQATVVAVGSGSK.G | 93 | 14 | gi\|4008131 | chaperonin 10 |

Supplementary table 2:

List of peptides identified by MS experiments after 2D separation of cell lysate proteins and spots excision.
