# Supplementary material for: Hsp10 nuclear localization and changes in lung cells response to cigarette smoke suggest novel roles for this chaperonin
Source: Open Biol. 2014 Oct 29;4(10):140125. doi: 10.1098/rsob.140125 (PMC4221893; doi:10.1098/rsob.140125)
Supplement: Supplementary Table 3 [file rsob140125supp3.docx]

**Supplementary table 3**

Prediction of nucleic acid-binding motifs in Hsp10^a^

| **Nuclei acid-binding motif** | **Hsp10** |
| --- | --- |
| DNA-binding motifs | None |
| RNA-binding motifs | None |

^a^Prediction method: PSORT II.
