# Supplementary material for: Hsp10 nuclear localization and changes in lung cells response to cigarette smoke suggest novel roles for this chaperonin
Source: Open Biol. 2014 Oct 29;4(10):140125. doi: 10.1098/rsob.140125 (PMC4221893; doi:10.1098/rsob.140125)
Supplement: Supplementary Table 4 [file rsob140125supp4.docx]

Supplementary table 4: Bioinformatic analysis of HSP10 interactors with known nuclear localization by searching the Biogrid (B)and IntAct (i) databases.

| **GENE** | **NAME** | **UniProt Id** | **DATABASE** |
| --- | --- | --- | --- |
| ATF2 | activating transcription factor 2 splice variant ATF2-var2 | P15336 | B |
| CBX3 | chromobox homolog 3 | Q13185 | B |
| CDK2 | Cyclin-dependent kinase 2 | P24941 | B |
| DCK | deoxynucleoside kinase | P27707 | B |
| EEF1A1 | eukaryotic translation elongation factor 1 alpha 1-like 14 | P68104 | B; I |
| EPB41 | erythrocyte membrane protein band 4.1 | P11171 | I |
| FBXO11 | F-box only protein 11 | Q86XK2 | I |
| FHIT | Bis(5'-adenosyl)-triphosphatase | P49789 | I |
| GBP2 | guanylate binding protein 2, interferon-inducible | P32456 | B; I |
| HSPA9 | heat shock 70kDa protein 9 (mortalin) | P38646 | B |
| IKBKE | Inhibitor of nuclear factor kappa-B kinase subunit epsilon | Q14164 | I |
| LRIF1 | ligand dependent nuclear receptor interacting factor 1 | Q5T3J3 | B |
| MDM2 | E3 ubiquitin-protein ligase Mdm2 | Q00987 | B |
| MPG | DNA-3-methyladenine glycosylase | P29372 | I |
| NRF1 | Nuclear respiratory factor 1 (alpha palindromic-binding protein) | Q16656 | B |
| RBBP7 | Histone-binding protein RBBP7 | Q16576 | B |
| RIF1 | Telomere-associated protein RIF1 | Q5UIP0 | I |
| TLE1 | transducin-like enhancer of split 1 | Q04724 | B; I |
| TRAF6 | E3 ubiquitin-protein ligase TRAF6 (TNF receptor-associated factor 6) | Q9Y4K3 | I |
| VHL | von Hippel-Lindau disease tumor suppressor | P40337 | I |
| ZHX1 | Zinc fingers and homeoboxes protein 1 | Q9UKY1 | B; I |
